# Epigenome-450K-wide methylation signatures of active cigarette smoking: The Young Finns Study

**DOI:** 10.1042/BSR20200596

**Published:** 2020-07-07

**Authors:** Pashupati P. Mishra, Ismo Hänninen, Emma Raitoharju, Saara Marttila, Binisha H. Mishra, Nina Mononen, Mika Kähönen, Mikko Hurme, Olli Raitakari, Petri Törönen, Liisa Holm, Terho Lehtimäki

**Affiliations:** 1 Department of Clinical Chemistry, Faculty of Medicine and Health Technology, Tampere University, Tampere, Finland; 2 Finnish Cardiovascular Research Center-Tampere, Faculty of Medicine and Health Technology, Tampere University, Tampere, Finland; 3 Department of Clinical Chemistry, Fimlab Laboratories, Tampere, Finland; 4 Gerontology Research Center (GEREC), Tampere University, Tampere, Finland; 5 Department of Clinical Physiology, Tampere University Hospital, Tampere, Finland; 6 Department of Microbiology and Immunology, Faculty of Medicine and Health Technology, Tampere University, Tampere, Finland; 7 Centre for Population Health Research, University of Turku and Turku University Hospital, Turku, Finland; 8 Research Centre of Applied and Preventive Cardiovascular Medicine, University of Turku, Turku, Finland; 9 Department of Clinical Physiology and Nuclear Medicine, Turku University Hospital, Turku, Finland; 10 Institute of Biotechnology, Helsinki Institute of Life Sciences (HiLife), University of Helsinki, Helsinki, Finland; 11 Organismal and Evolutionary Biology Research Program, Faculty of Biological and Environmental Sciences, University of Helsinki, Helsinki, Finland

**Keywords:** epigenomics, Metylation, Tobacco smoke

## Abstract

Smoking as a major risk factor for morbidity affects numerous regulatory systems of the human body including DNA methylation. Most of the previous studies with genome-wide methylation data are based on conventional association analysis and earliest threshold-based gene set analysis that lacks sensitivity to be able to reveal all the relevant effects of smoking. The aim of the present study was to investigate the impact of active smoking on DNA methylation at three biological levels: 5′-C-phosphate-G-3′ (CpG) sites, genes and functionally related genes (gene sets). Gene set analysis was done with mGSZ, a modern threshold-free method previously developed by us that utilizes all the genes in the experiment and their differential methylation scores. Application of such method in DNA methylation study is novel. Epigenome-wide methylation levels were profiled from Young Finns Study (YFS) participants’ whole blood from 2011 follow-up using Illumina Infinium HumanMethylation450 BeadChips. We identified three novel smoking related CpG sites and replicated 57 of the previously identified ones. We found that smoking is associated with hypomethylation in shore (genomic regions 0–2 kilobases from CpG island). We identified smoking related methylation changes in 13 gene sets with false discovery rate (FDR) ≤ 0.05, among which is olfactory receptor activity, the flagship novel finding of the present study. Overall, we extended the current knowledge by identifying: (i) three novel smoking related CpG sites, (ii) similar effects as aging on average methylation in shore, and (iii) a novel finding that olfactory receptor activity pathway responds to tobacco smoke and toxin exposure through epigenetic mechanisms.

## Background

Tobacco use has been estimated to cause approximately 7 million yearly deaths globally through general morbidity, disability, non-communicable chronic diseases, and increased risk of communicable diseases [1]. The chronic diseases caused by smoking are in nature multifactorial, mediated by both genetic and environmental factors [2,3]. Despite the well-established link between smoking and associated diseases in literature, there is a knowledge gap in the causative mechanisms of the diseases.

Previous studies have led to identification of several transcriptomic signatures and their role in diseases related to smoking [4]. Epigenetic mechanisms have been understood to be modulated by environmental factors such as smoking and play a crucial role in disease development process by regulating gene expression [5]. Several studies have reported smoking-related methylation changes at CpG site, gene as well as gene set level [6–11]. Zeilinger et al. reported 972 CpG sites significantly associated with smoking from discovery data with 1793 participants and replicated 187 out of 972 CpG sites in independent cohort of 479 participants [6]. The three most significant CpG sites and corresponding genes were *cg05575921:AHRR, cg21566642: ALPP/ALPPL2*, and *cg03636183:F2RL3*. McCartney et al. [7] and Zeilinger et al. [6] suggested that smoking-induced DNA methylation changes are reversible. Shenker et al. developed methylation index by combining four smoking related CpG sites (one in AHRR, two intergenic in chromosome 2 and one intergenic in chromosome 6) that can predict former smoking status [8]. The study by Joehanes et al., one of the largest study of smoking-related DNA methylation changes, identified 18760 active smoking-related CpG sites annotated to 7201 genes with FDR < 0.05 [9]. Prince et al. investigated association of 2620 previously reported smoking-related CpG sites with different smoking behaviors (such as ever smoking, current weekly smoking, ever weekly smoking, and blood cotinine levels) and reported smoking behavior related methylation patterns in 11 CpG sites mapping to seven genes [10]. Several studies on impact of aging on DNA methylation, as summarized by Ciccarone et al., have established age-induced DNA methylation changes as hallmark of aging [12]. Recent studies such as by Yang et al. [13] and Lei et al. [14] have reported association between cigarette smoking and age-induced DNA methylation changes. Joehanes et al. also performed Gene Ontology (GO) [15] based gene set analysis of the list of statistically significant CpG sites and reported 99 gene sets that broadly included molecular processes such as signal transduction, protein metabolic process, and transcription pathways [9]. Another study performed gene set analysis of list of CpG sites with altered methylation levels due to exposure to maternal smoking during pregnancy and reported cell cycle, cancer, white blood cell differentiation, genotoxicity as major findings [16]. Bakulski et al. performed gene set analysis of lung-specific smoking related CpG sites and the most significant pathways included mRNA catabolic processes, protein targeting, angiogenesis, and mRNA translation [17].

Traditional epigenome-wide association studies might fail to identify relevant CpG sites due to noise and lack of statistical power. Further, long list of CpG sites discovered through such studies are harder to interpret in terms of underlying biological theme. Gene set analysis, also referred to as pathway analysis, addresses the problems by shifting the analysis from individual CpG sites to groups of related CpG sites. CpG sites are mapped to respective genes and grouped together based on shared biological features. The grouping of corresponding genes can be based on reference knowledge bases such as Reactome Pathway Database [18], BioCarta pathways [19], Kyoto Encyclopedia of Genes and Genomes (KEGG) [20], and GO [15]. The grouping approach averages out errors at single CpG site level and increases statistical power. Additionally, the outcome of gene set analysis is meaningful terms that provide insights into biological theme underlying the list of CpG sites.

Studies that have reported smoking-related methylation changes at biological pathway level have done so mostly with the earliest gene set analysis approach, over-representation analysis (ORA) [9,17,21]. ORA is a type of gene set analysis that is based on statistical tests like hypergeometric and chi-square. The approach requires users to provide a list of genes selected based on arbitrary threshold (for example, *P*-value<0.05). The arbitrariness of the threshold leads to unstable results that are difficult to replicate and massive reduction in sensitivity. Furthermore, the approach assumes independence between genes, which is violated in most cases resulting to false positive results [22].

Over a decade of development of gene set analysis approaches have provided several robust gene set analysis methods that are likely to provide improved insights into epigenetic consequences of smoking. A class of such methods includes threshold-free competitive gene set analysis methods that test whether genes in a given gene set are more differentially methylated than the other genes in the dataset. The approach can be used to test a large number of gene sets, such as those provided by Molecular Signatures Database (MSigDB) [23,24] in order to profile smoking-related epigenetic alterations at biological pathways or processes level. An example of one of such methods is *mGSZ* (modified Gene Set *Z*-score) that have been developed and improved over time by us [25–27] and have been shown to be robust and efficient by us [20–22] as well as another independent study [28]. Unlike over-representation analysis methods, *mGSZ* is threshold-free and thus analyzes all the genes in the data resulting into sensitive, comprehensive, and reproducible results. The *mGSZ* is different from other methods in the class in that it is based on asymptotic *P*-value estimation that is significantly more efficient than empirical *P*-value estimation [26,29].

The objective of the present study was to characterize DNA methylation differences between current smokers and non-smokers at different biological levels such as: (1) CpG site level with differential methylation analysis; (2) biological pathway level using a threshold-free gene set analysis method, *mGSZ*; and (3) average methylation level at different genomic regions with Wilcoxon rank-sum test. We show that application of threshold-free gene set analysis method for DNA methylation data has improved benefit over earliest threshold-based ORA methods used currently by most studies as illustrated by our novel findings such as smoking-induced altered methylation in olfactory system. Even though olfactory ability is known to be adversely affected by active cigarette smoking [30], we for the first time show the underlying epigenetic mechanism.

## Materials and methods

### Study population

Young Finns Study (YFS) is one of the largest existing follow-up studies into cardiovascular health from childhood to adulthood, running in a longitudinal prospective setup with regular follow-ups from 1980 onwards [31]. The study began in 1980 with 3596 children and adolescents aged 3 to 18 years randomly selected from the areas of five university hospitals in Finland (Turku, Tampere, Helsinki, Kuopio, and Oulu). The participants have been followed up for over 40 years. The methylation measurements were performed on a subset of 192 individuals from whole-blood samples from 2011 follow up. The smoking history of the subjects was self-reported and belonged to six categories based on smoking frequency (1. active smoker or at least once a day, 2. once a week or more often, however not daily, 3. less often than once a week, 4. attempts to quit, 5. has quit, 6. has never smoked). The present study is based on a subsample of 125 participants, 40–49 years of age who were either active smoker (*n*=21) or have never smoked (*n*=104) [Table 1]. Participants in the middle conditions such as those who smoked less frequently or has quit smoking were eliminated in order to obtain maximum possible smoking-related biological signal in DNA methylation.

**Table 1:** Population characteristics of Young Finns Study participants; data are mean ± SD or proportions

Characteristics	Active smokers	Non-smokers
Number of subjects	21	104
Sex (%men)	57%	34%
Age, years	45 (±3.95)	44.1 (±3.1)
Body mass index, kg/m^2^	26.59 (±4.38)	25.7 (±4.36)

### DNA methylation assessment

DNA was extracted from EDTA-blood samples using Wizard®Genomic DNA Purification Kit (Promega Corporation, Madison, WI, U.S.A.), according to the manufacturer’s instructions. Genome-wide quantification of DNA methylation levels were done using Illumina Infinium HumanMethylation450 BeadChips [32] in the Core Facility at the Institute of Molecular Medicine Finland, University of Helsinki, following manufacturer’s protocols. The HumanMethylation450 BeadChip measures DNA methylation at more than 485,000 CpG cites across the genome. The arrays were imaged with a high-precision scanner (iScan system, Illumina Inc.), and the signal intensities were extracted using a GenomeStudio software package (Illumina Inc.). The methylation data are available in Gene Expression Omnibus (GEO) under accession number GSE69270.

### Data filtering and normalization

Data were obtained and processed from raw methylation image files using the *minfi* package in R/Bioconductor [33]. All the analyzed samples have sum of detection *P*-values across all the probes less than 0.05. Logged (log2) median of methylated and unmethylated intensities of the analyzed samples clustered within the default threshold (10.5) of *getQC* function in *minfi*. Further, samples for which real sex did not match the predicted sex obtained with *getSex* function in *minfi* were excluded. Background subtraction and dye-bias normalization were performed via noob method [34] implemented in *minfi*. Stratified quantile normalization was performed using *preprocessQuantile* function in *minfi*. Probes with detection *P*-value more than 0.01 in 99% of the samples were filtered out. CpG loci on sex chromosomes were excluded from the analysis to avoid gender-based methylation bias. Also, cross-reactive probes and probes with SNPs were excluded from the analysis. After quality control, the total number of autosomal CpGs was 429,773 in 125 samples (21 active smokers and 104 non-smokers). Batch correction was done by adding the first two principal components of control probes as covariates in multiple linear regression model.

### Differential methylation analysis

All statistical analyses were performed using R statistical software (v.3.5.1) [35]. *M*-values, calculated as the log2 ratio of the intensities of methylated probe versus unmethylated probe were used as measures of methylation level. Differentially methylated CpG loci for smoking status were identified using *CpGassoc* package in R [36]. In order to keep the present study exploratory in nature, analyses were based on two different models adjusted with varying number of covariates. Model 1 involved adjustment for age, sex, body mass index, cell type proportions, and technical covariates (chip and array). Cell type proportions consisted of proportions of CD8T, CD4T, natural killer cells, B cells, monocytes, and granulocytes in white blood cells. Selection of covariates for model 1 was motivated from study by [9]. Model 2 involved adjustment with two additional covariates, alcohol usage and socioeconomic status, in addition to those in model 1. The addition of the two covariates in model 2 is based on previous reports suggesting effects of alcohol usage [37] and socioeconomic status [38] on DNA methylation. There was reduction in sample size for model 2 because 16 out of 125 participants did not have information on alcohol usage and socioeconomic status. The number of active smokers in the analysis was reduced from 21 to 16 because of missing information on socioeconomic status. Alcohol consumption was measured by asking participants to report their alcohol consumption during the previous week. One unit is equivalent to 14 g of alcohol [39]. Socioeconomic status was based on occupation and was categorized as manual, lower non-manual, and upper non-manual. Potential batch affects were addressed by including the first two principal components of array control probes into the regression models. The cell type proportions of white blood cells were estimated through the reference-based Houseman method [40] using the *estimateCellCounts* function in the *minfi* Bioconductor package in R. CpG sites were mapped to genes by using annotation database provided by Illumina [41]. Differentially methylated genes were identified by utilizing as a proxy the CpG site with maximum absolute *t*-score from any location in the genomic region of the gene, since the mechanism how the methylation influences gene expression is not completely understood. Statistical significance level was set to false discovery rate (FDR) of 0.05 in the CpG site and gene level analysis.

### Gene set analysis

Biological relevance of the differentially methylated genes based on both model 1 and 2 was investigated using *mGSZ* method implemented in *mGSZ* R package [26]. mGSZ is a gene set analysis method based on robust gene set scoring function and efficient *P*-value estimation method. Unlike over-representation-based pathway analysis methods [42,43], this approach is threshold free and thus includes all the genes in the analysis irrespective of their effect size or significance level. This is particularly important in the present study as our goal is to identify set of genes contributing to a biological pathway that have milder but coherent smoking-related changes in methylation level. Analyzed gene sets were downloaded from MSigDB version 7.0. The database contained 22569 gene sets (as of September 21, 2019) divided into eight major collections: Hallmark gene sets, positional gene sets, curated gene sets, motif gene sets, computational gene sets, gene ontology (GO) gene sets, oncogenic signatures, and immunogenic signatures. Hallmark gene sets are generated computationally and represent well-defined biological processes [44]. Positional gene sets are generated based on genomic locations of genes. Curated gene sets are generated from knowledge sources such as pathway databases, biomedical literature, and domain experts. The gene sets are divided into two sub-categories: (i) canonical pathways curated from online databases [18–20] and (ii) chemical and genetic perturbations (CGP). CGP-based gene sets represent expression signatures of chemical and genetic perturbations mostly curated from biomedical literature [45]. Motif gene sets represent target genes for transcription factors or micro RNAs. Computational gene sets are generated from cancer related microarray gene expression data with data mining techniques. GO gene sets are derived from GO annotations and have three sub-categories—biological process (BP), molecular function (MF), and cellular component (CC).

### Average methylation level at different genomic regions

We analyzed effect of smoking on the average methylation level at different genomic regions in relation to both gene region (TSS200, 0–200 bases upstream of the transcriptional start site; TSS1500, 200–1500 bases upstream of the TSS; 5′UTR, within the 5′ untranslated region, between the TSS and the ATG start site; Body, between the ATG and stop codon irrespective of the presence of introns, exons, TSS, or promoters; 3′UTR, between the stop codon and poly A signal) and CpG islands (Shores, 0–2 kb from CpG island; Shelves, 2–4 kb from CpG island). The information about the different genomic regions of CpG sites on HumanMethylation450K array was obtained from Illumina Inc. Mean of methylation β-values of all CpGs belonging to the genomic regions for each of the study participants was calculated. Smoking-related statistical difference in average methylation specific to each of the regions was tested with Wilcoxon rank-sum test.

## Results

### Study participants’ characteristics

Characteristics of the YFS cohort participants in the present study is summarized in Table 1.

### Differential methylation analysis

**Figure 1:**
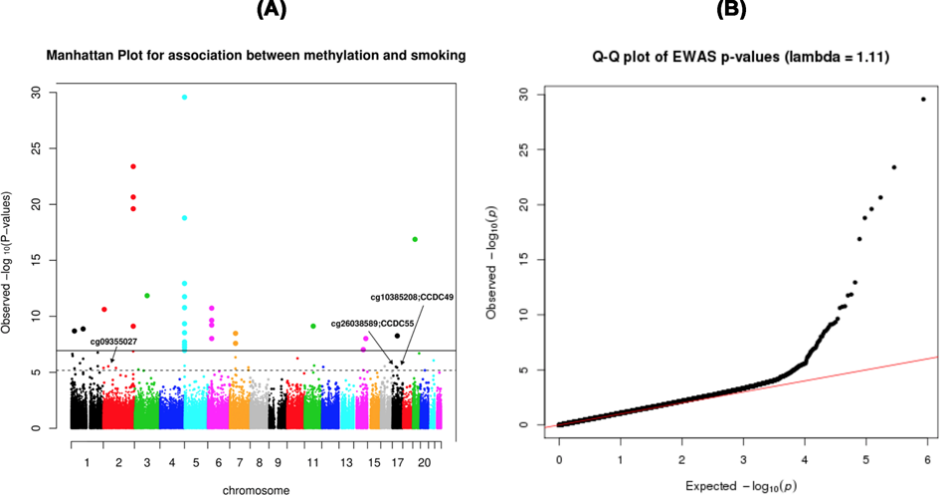
Manhattan plot and quantile-quantile (Q-Q) plot of epigenome-wide association analysis of smoking habit (**A**) Manhattan plot showing the *P*-values of genome-wide CpG sites. *X*-axis represents position of the CpG sites on each chromosome. *Y*-axis represents negative log10 of the *P*-values for the association. The dotted line indicates false discovery rate (FDR)-corrected significance threshold and the solid horizontal line represents Bonferroni-corrected significance threshold (experiment-wide significance). The annotations for the three novel CpG sites and corresponding genes (if annotation is known) are shown. (**B**) Quantile-quantile plot showing genomic inflation factor (lambda = 1.11) of the epigenome-wide association study. The genomic inflation factor (ratio of the median of the empirically observed distribution of the test statistic to the expected median) represents the extent of inflation and false positive rate in the results.

We identified 60 differentially methylated CpG sites with respect to smoking habit with FDR < 0.05 from statistical model 1 [Table 2]. There were 26602 nominally significant (*P*-value < 0.05) CpG sites (Figure 1). The results identify three novel smoking-associated CpG sites: cg26038589 (CCDC55, Coiled-Coil Domain-Containing Protein 55), cg10385208 (CWC25, Spliceosome Associated Protein Homolog), and cg09355027 (no known gene) and replicate the findings of the previous studies, indicating that our results are technically robust. In addition, a site found in only one previous study, cg13898430 (RUNX3, Runt-related transcription factor 3) [46] was also replicated in the present study. Model 2 identified only 18 differentially methylated CpG sites with respect to smoking habit with FDR < 0.05. Seventeen of the CpG sites from model 2 that were also reported by model 1 are highlighted in Table 2 with bold font. The three novel sites identified in model 1 were not recovered with model 2.

**Table 2:** List of differentially methylated CpG sites with respect to smoking habit with false discovery rate (FDR) < 0.05 identified with model 1, their corresponding effect size, standard error, *P*-values, FDR, and genomic regions in relation to both gene region (TSS200, 0–200 bases upstream of the transcriptional start site; TSS1500, 200–1500 bases upstream of the TSS; 5′UTR, within the 5′ untranslated region, between the TSS and the ATG start site; Body, between the ATG and stop codon irrespective of the presence of introns, exons, TSS, or promoters; 3′UTR, between the stop codon and poly A signal) and CpG islands (Shores, 0–2 kb from CpG island; Shelves, 2–4 kb from CpG island)

	CpG Sites	Effect size	Standard error	*P*-value	FDR	Gene/Island name	Gene region/Relation to island	Chromosome
1	cg20295214	0.03	0.005	9.53 × 10^-9^	1.84 × 10^-4^	AVPR1B	Body	1
2	cg08709672	0.02	0.004	3.21 × 10^-6^	2.82 × 10^-2^	AVPR1B	5′ UTR 1stExon	1
3	cg09935388	0.06	0.009	9.87 × 10^-10^	2.65 × 10^-5^	GFI1	Body	1
4	cg18316974	0.03	0.006	7.64 × 10^-7^	8.87 × 10^-3^	GFI1	Body	1
5	cg18146737	0.05	0.01	5.55 × 10^-6^	4.18 × 10^-2^	GFI1	Body	1
6	cg25189904	0.03	0.006	3.85 × 10^-6^	3.18 × 10^-2^	GNG12	TSS1500	1
7	**cg21408581**	0.02	0.005	2.18 × 10^-6^	2.03 × 10^-2^	RAB3GAP2	Body	1
8	cg08869700	0.01	0.003	2.75 × 10^-6^	2.46 × 10^-2^	RFX5	Body	1
9	cg13898430	0.008	0.002	5.07 × 10^-6^	3.96 × 10^-2^	RUNX3	TSS1500	1
10	cg04885881	0.02	0.004	1.52 × 10^-7^	2.43 × 10^-3^	chr1:11119030 -11120634	Shelf	1
11	cg27537125	0.02	0.003	9.85 × 10^-9^	1.84 × 10^-4^	–	OpenSea	1
12	cg12547807	0.01	0.002	6.36 × 10^-7^	7.84 × 10^-3^	–	OpenSea	1
13	cg11555067	0.02	0.003	3.42 × 10^-6^	2.94 × 10^-2^	INPP4A	5′ UTR	2
14	**cg21566642**	0.03	0.002	1.02 × 10^-23^	2.19 × 10^-18^	chr2:233283397 -233285959	Island	2
15	**cg01940273**	0.05	0.004	2.46 × 10^-21^	3.53 × 10^-16^	chr2:233283397 -233285959	Island	2
16	**cg05951221**	0.05	0.004	8.74 × 10^-19^	7.51 × 10^-14^	chr2:233283397 -233285959	Island	2
17	cg03329539	0.02	0.003	2.71 × 10^-9^	6.86 × 10^-5^	chr2:233283397 -233285959	Shore	2
18	cg06644428	0.06	0.01	9.64 × 10^-7^	1.09 × 10^-2^	chr2:233283397 -233285959	Island	2
19	cg21949194	0.02	0.003	6.06 × 10^-6^	4.49 × 10^-2^	chr2:39351355 -39351733	Shelf	2
20	cg23079012	0.02	0.003	5.43 × 10^-10^	1.67 × 10^-5^	–	OpenSea	2
21	cg09355027 (**novel**)	0.02	0.004	1.43 × 10^-6^	1.46 × 10^-2^	–	OpenSea	2
22	**cg19859270**	0.03	0.004	9.59 × 10^-12^	3.75 × 10^-7^	GPR15	1st Exon	3
23	cg24719910	0.02	0.003	5.41 × 10^-6^	4.15 × 10^-2^	TGFBR2	Body	3
24	**cg05575921**	0.10	0.006	8.53 × 10^-28^	3.66 × 10^-22^	AHRR	Body	5
25	**cg21161138**	0.04	0.004	1.09 × 10^-19^	1.17 × 10^-14^	AHRR	Body	5
26	**cg25648203**	0.04	0.004	5.82 × 10^-13^	3.57 × 10^-8^	AHRR	Body	5
27	**cg14817490**	0.05	0.005	2.01 × 10^-12^	9.60 × 10^-8^	AHRR	Body	5
28	**cg26703534**	0.03	0.003	4.53 × 10^-12^	1.95 × 10^-7^	AHRR	Body	5
29	**cg24090911**	0.03	0.005	1.38 × 10^-10^	4.56 × 10^-6^	AHRR	Body	5
30	cg12806681	0.04	0.006	1.44 × 10^-8^	2.59 × 10^-4^	AHRR	Body	5
31	cg03991871	0.05	0.009	1.12 × 10^-7^	1.85 × 10^-3^	AHRR	Body	5
32	cg04551776	0.02	0.004	1.68 × 10^-7^	2.58 × 10^-3^	AHRR	Body	5
33	cg23916896	0.05	0.009	3.73 × 10^-7^	5.17 × 10^-3^	AHRR	Body	5
34	cg11554391	0.02	0.004	1.21 × 10^-6^	1.27 × 10^-2^	AHRR	Body	5
35	cg01899089	0.02	0.003	4.16 × 10^-6^	3.31 × 10^-2^	AHRR	Body	5
36	cg11902777	0.05	0.009	6.94 × 10^-6^	4.97 × 10^-2^	AHRR	Body	5
37	**cg06126421**	0.06	0.007	1.04 × 10^-12^	5.61 × 10^-8^	–	OpenSea	6
38	**cg24859433**	0.03	0.004	6.12 × 10^-11^	2.19 × 10^-6^	–	OpenSea	6
39	cg14753356	0.02	0.003	3.15 × 10^-9^	7.52 × 10^-5^	–	OpenSea	6
40	**cg15342087**	0.02	0.003	6.49 × 10^-9^	1.39 × 10^-4^	–	OpenSea	6
41	cg12803068	-0.09	0.01	2.41 × 10^-7^	3.45 × 10^-3^	MYO1G	Body	7
42	cg04180046	-0.03	0.005	6.39 × 10^-7^	7.84 × 10^-3^	MYO1G	Body	7
43	cg22132788	-0.05	0.007	1.58 × 10^-6^	1.58 × 10^-2^	MYO1G	Body	7
44	cg07826859	0.01	0.003	1.94 × 10^-6^	1.89 × 10^-2^	MYO1G	TSS1500	7
45	cg21322436	0.01	0.003	4.06 × 10^-6^	3.29 × 10^-2^	CNTNAP2	TSS1500	7
46	cg03450842	0.01	0.003	4.42 × 10^-7^	5.93 × 10^-3^	ZMIZ1	5′ UTR	10
47	cg01901332	0.02	0.004	1.19 × 10^-6^	1.27 × 10^-2^	ARRB1	Body	11
48	cg21611682	0.02	0.003	7.56 × 10^-10^	2.17 × 10^-5^	LRP5	Body	11
49	cg14624207	0.01	0.003	6.91 × 10^-6^	4.97 × 10^-2^	LRP5	Body	11
50	cg07986378	0.02	0.005	2.49 × 10^-6^	2.28 × 10^-2^	ETV6	Body	12
51	**cg22851561**	0.02	0.003	3.90 × 10^-8^	6.71 × 10^-4^	C14orf43	5′ UTR	14
52	**cg05284742**	0.02	0.003	4.53 × 10^-9^	1.03 × 10^-4^	ITPK1	Body	14
53	cg07069636	0.01	0.002	4.82 × 10^-7^	6.28 × 10^-3^	chr16:30669107 -30671155	Shore	16
54	cg26038589 (**novel**)	0.03	0.006	1.17 × 10^-6^	1.27 × 10^-2^	CCDC55	Body	17
55	cg10385208 (**novel**)	0.01	0.003	3.81 × 10^-6^	3.18 × 10^-2^	CWC25	TSS1500	17
56	cg19572487	0.02	0.004	8.07 × 10^-9^	1.65 × 10^-4^	RARA	5′ UTR	17
57	**cg03636183**	0.05	0.004	1.00 × 10^-17^	7.17 × 10^-13^	F2RL3	Body	19
58	cg03707168	0.02	0.004	7.10 × 10^-7^	8.47 × 10^-3^	PPP1R15A	Body	19
59	cg17566560	0.02	0.004	2.13 × 10^-6^	2.03 × 10^-2^	DLGAP4	Body, 5′UTR	20
60	cg23110422	0.03	0.006	1.85 × 10^-7^	2.74 × 10^-3^	ETS2	Body	21

Intergenic chromatin regions are called OpenSea. CpG sites retained in model 2 are highlighted with bold font. **Statistical models:** Model 1 involved adjustment for age, sex, body mass index, cell type proportions, and technical covariates (chip and array). Model 2 involved adjustment with two additional covariates, alcohol usage and socioeconomic status, in addition to those in model 1.

### Gene set analysis

**Figure 2:**
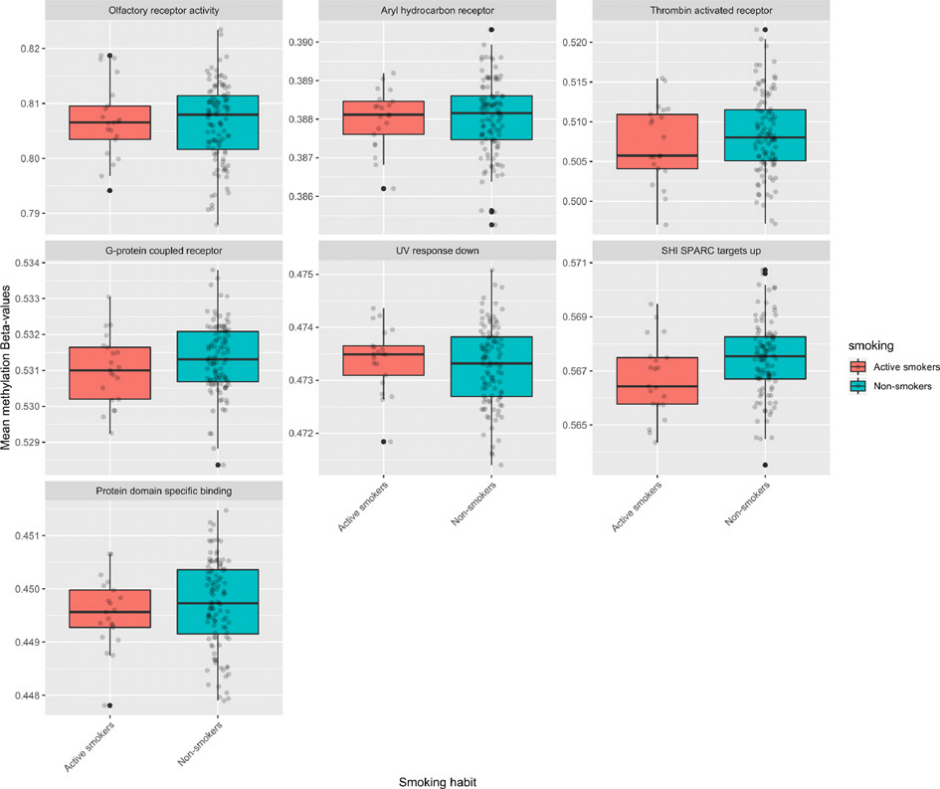
Box plots showing differences in average methylation of all CpG sites belonging to genes of the analyzed gene sets in smokers and non-smokers *X*-axis represents smoking habit (active smokers and non-smokers). *Y*-axis represents mean methylation β-values of CpG sites belonging to genes of the analyzed gene sets.

Using the gene sets from MSigDB and *mGSZ*, we identified smoking induced altered methylation in a total of 13 gene sets with FDR ≤ 0.05. The results are presented in Table 3 and Figure 2. The figure serves the purpose of observing overall methylation pattern and we do not expect significant statistical differences with such approach due to sample size limitation. This is where our proposed state-of-the-art gene set analysis method, *mGSZ*, becomes useful as the approach increases the statistical power and identifies biologically relevant results even with smaller sample size.

**Table 3:** Molecular Signature Database based gene sets identified with false discovery rate (FDR) ≤ 0.05 in the present study

	Significant gene sets	Size (number of genes) of gene sets	GSZ-score	*P*-value	FDR
	***Gene Ontology (GO) based gene sets***				
1.	Regulation of smoothened signaling pathway involved in dorsal ventral neural tube (biological process)	8	4.89	5.12 × 10^-6^	0.04
2.	Thrombin-activated receptor activity (molecular function)	5	4.27	2.3 × 10^-5^	0.03
3.	Protein domain specific binding (molecular function)	657	4.75	5.7 × 10^-5^	0.03
4.	Olfactory receptor activity (molecular function)	321	9.96	6.09 × 10^-5^	0.03
5.	Semaphorin receptor activity (molecular function)	9	4	6.85 × 10^-5^	0.03
6.	AP-2 adaptor complex binding (molecular function)	7	4.22	1.15 × 10^-4^	0.03
7.	Nuclear receptor activity (molecular function)	45	4.81	1.18 × 10^-4^	0.03
8.	G-protein coupled receptor activity (molecular function)	725	7.21	2.28 × 10^-4^	0.05
	***Curated gene sets***				
9.	Aryl hydrocarbon receptor signaling (canonical, reactome)	7	5.76	6.27 × 10^-7^	0.003
10.	SHI SPARC targets up (chemical and genetic perturbations)	22	5.86	1.22 × 10^-6^	0.003
11.	RORA activates gene expression (canonical, reactome)	17	4.99	1.68 × 10^-5^	0.03
12.	RARRXR pathway (canonical, biocarta)	7	4.38	3.41 × 10^-5^	0.05
	***Hallmark gene sets***				
13.	UV response down	138	4.50	0.001	0.05

Among eight major gene set collections of MSigDB, significant results (FDR ≤ 0.05) were obtained for three—Hallmark, Curated, and GO gene sets [Table 3]. The gene set, *Genes down-regulated in response to ultraviolet (UV) radiation* (FDR = 0.05) identified from Hallmark collection suggests that smoking induces hypermethylation of genes that are known to be down-regulated by ultraviolet radiation [Table 3 and Figure 2]. We identified three canonical pathways with smoking related altered methylation (hypomethylation): two from reactome database and one from BioCarta. Gene sets based on reactome pathways are *aryl hydrocarbon receptor signaling* (FDR = 0.003) and *rora activates gene expression* (FDR = 0.03). Similarly, BioCarta based results suggest that smoking alters methylation in genomic region responsible for RARRXR pathway (FDR = 0.05), which is linked to cancer. CGP-based gene set identified in the present study suggests altered methylation in genes that are up-regulated in glioma cell lines after knockdown of SPARC gene by RNAi (FDR = 0.003). GO annotation-based gene sets were analyzed separately for the three GO categories. We identified one BP and 7 MF related gene sets with FDR ≤ 0.05 [Table 3]. Olfactory receptor activity, a novel finding of this study, belongs to GO MF molecular.

Gene set analysis of differentially methylated CpG sites identified with model 2 reported seven gene sets with FDR < 0.25 [Supplementary Table S2]. The novel finding of the present study, smoking-induced altered methylation in olfactory system, was retained with FDR of 0.14. Also, the well-established effect of smoking on aryl hydrocarbon receptor signaling was retained with FDR of 0.004. Similarly, effect of smoking on UV sensitive genes as well as four other gene sets with gene regulatory effect was retained.

### Average methylation level analysis specific to different genomic regions

**Figure 3:**
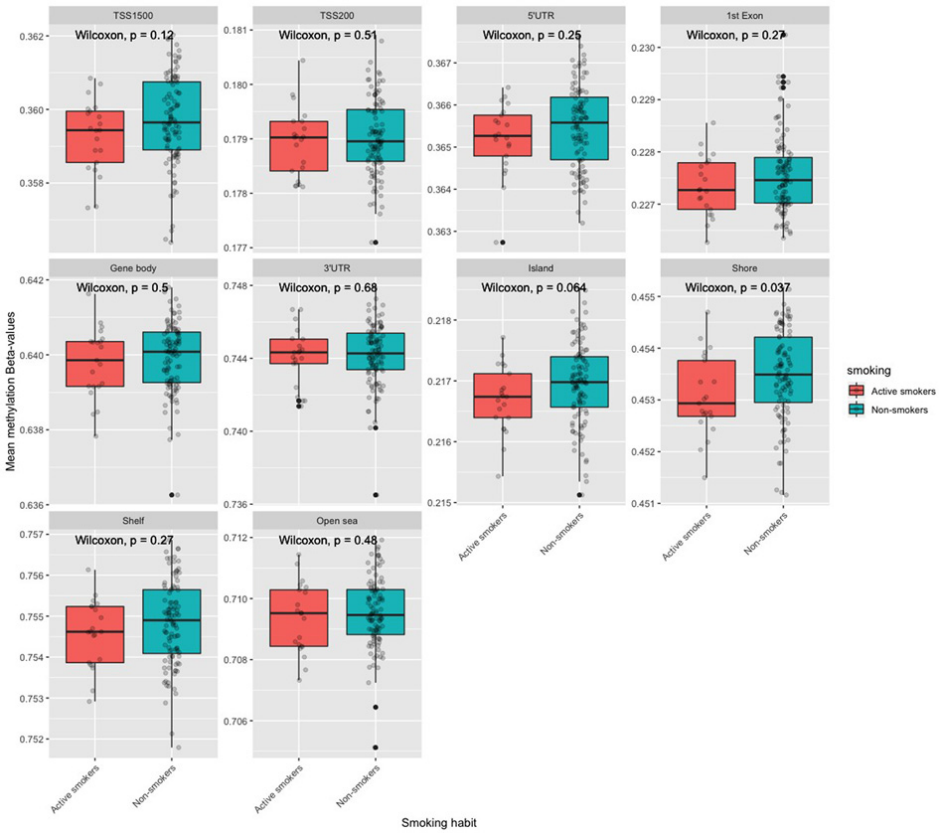
Effect of smoking on average methylation level in different genomic regions in our genome Box plots showing smoking-related differences in average methylation levels in different genomic regions. *X*-axis represents smoking habit (active smokers and non-smokers). *Y*-axis represents average methylation β values of CpG sites belonging to different genomic regions. **Abbreviations and definitions for gene regions**: TSS200, 0–200 bases upstream of the transcriptional start site; TSS1500, 200–1500 bases upstream of the TSS; 5′UTR, within the 5′ untranslated region, between the TSS and the ATG start site; Body, between the ATG and stop codon irrespective of the presence of introns, exons, TSS, or promoters; 3′UTR, between the stop codon and poly A signal. **Definitions for CpG islands**: Shores, 0–2 kb from CpG island; Shelves, 2–4 kb from CpG island.

We found that active smoking is significantly (Wilcoxon rank-sum test, *P*-value < 0.05) associated with hypomethylation in shore region among active smokers [Supplementary Table S1 and Figure 3]. Although the conventional *P*-value threshold of 0.05 was not reached for other CpG regions, perhaps due to lack of power, smoking seems to be associated with hypomethylation in most of the regions [Supplementary Table S1]. These results suggest that effect of active smoking on average methylation in genomic regions such as shore resembles to that of aging [12].

## Discussion

To the best of our knowledge, this is the first study that implements a modern threshold free gene set analysis method to study smoking-induced alterations in methylation in genomic regions at biological pathway level. Studies of biological implications of CpG sites with altered methylation due to smoking have been limited to traditional over-representation-based pathway analysis [9,17,21,47]. We performed differential methylation analysis to first identify CpG sites that are significantly associated with smoking habit. We then implemented our robust gene set analysis method, *mGSZ*, on the whole methylation data at gene level to identify biological processes that might be affected due to smoking-induced altered methylation. The present study extended the current knowledge by identifying three novel smoking-associated CpG sites and altered methylation in genomic regions that play role in olfactory sensing system, immune response, cardiovascular disease, and cancer development. Furthermore, the results suggest that the global effect of smoking is similar to aging process that is known to be associated with hypomethylation in genomic regions with high frequency of CpG sites such as shores [12]. Hypomethylation in the genomic regions of active smokers is in line with findings at CpG site and gene set level. All except three CpG sites in the region of MYO1G gene are hypomethylated among active smokers. Similarly six out of seven significant gene sets based on model 2 are hypomethylated among active smokers.

Previously, unidentified site cg26038589 maps to gene CCDC55. The protein product of the gene plays role in integrated regulation of gene expression in various ways and contribute to the development of cancers, viral infections, and neurological disorders [48]. CpG site cg10385208 is located in the genomic region of gene CWC25. This gene has been annotated to be overexpressed in lung and peripheral blood mononuclear cells in GeneCards—the human gene database [49]. The annotation is based on analysis of 69 proteomics datasets from Human Integrated Protein Expression Database resided within GeneCards. The gene has also been shown to play role in cognitive impairment [50]. Furthermore, the genes CCDC55 and CWC25 are linked to alternative splicing suggesting a role of DNA methylation on alternative splicing. The genes, therefore, should be interesting targets to follow up in the laboratory. The third novel site, cg09355027 has not been annotated to any gene and its biological significance is unknown. However, the CpG site is one of the numerous sites whose methylation is modulated by methyl-CpG-binding domain protein 2 (MBD2) [51]. MBD2 participates in gene silencing and has active role in shaping the cancer methylome. Site cg13898430 identified in our study has been identified previously to be associated with smoking by only one study [46]. The CpG site maps to gene RUNX3, a runt domain-containing transcription factor and a known tumor suppressor, frequently deleted or transcriptionally silenced in cancer according to the NCBI Gene database [52]. The gene is associated with numerous cancers, including lung cancer and several autoimmune diseases, including the inflammatory bowel disease. Overall, both the replicated and the discovered methylation sites map to genes that play roles in biological processes and pathways plausibly connected to smoking-induced disease.

Olfactory receptor activity, one of the significant gene sets identified in the present study, is responsible for initiating cell activity in response to smell detection. Surprisingly, despite the long-established knowledge that smoking is associated with olfactory and gustatory dysfunction, relevant pathways have not been reported in previous studies. Decline in the sense of smell has been proposed to originate from several mechanisms operating in parallel, including the tobacco-induced sinonasal inflammation and squamous cell metaplasia in the airways [53]. The olfactory epithelium is a highly dynamic structure going through constant renewal through the exfoliation of the aged cells and the generation of new ones from the stem cells, thus constantly regenerating the sensory neurons [54]. The division and differentiation of the stem cells are regulated by conserved epigenetic mechanisms, including DNA methylation [55]. As the cycle is constantly repeated, it is conceivable that the tobacco smoke exposure effects would be seen widely. In our study, these effects appear to be reflected indirectly in the epigenome measured from the blood. It is noteworthy that the genes related to olfactory system did not reach statistical significance threshold in the gene level differential methylation analysis, while their co-occurrence as a gene set (on pathway level) passes statistical significance threshold. This could partially explain why the olfactory pathways have been missed by many previous studies utilizing only the highest ranking genes in over-representation-based gene set analysis.

Consistent with the previous findings that tobacco exposure leads to neural tube defects, the present study identified altered methylation in genes that play role in regulation of smoothened signaling pathway involved in dorsal ventral neural tube [56]. Gene sets representing *semaphoring receptor activity* and *G-protein–coupled receptor* identified in our study have been annotated to the immune system. Altered methylation of these receptors due to smoking might deteriorate regulation of immunity [57]. We also identified altered methylation in genes involved in the thrombin signaling pathway that suggests that smoking plays role in mechanisms of coronary thrombosis [58]. Gene sets based on *aryl hydrocarbon receptor signaling, RORA activates gene expression*, and *RARRXR pathway* identified in our study are related to gene regulation and consequently have a role in cancer. Similarly, we identified a set of genes in glioma cells that are up-regulated after knockdown of SPARC (secreted protein acidic and cysteine rich) by RNAi. Previous study has shown that nicotine is associated with stimulation of malignant behavior of glioma cells [59]. To summarize, in addition to the novel findings such as smoking-induced alteration in methylation in olfactory system and ultraviolet radiation sensitivity genes, the previously identified biological processes and pathways in cardiovascular disease, immune response, and cancer were prominent in our study.

The main weaknesses of the present study are the small sample size, self-reported smoking status and alcohol usage information, and occupation-based SES assessment. However, nearly all of the significant hits replicated the previous findings indicating the robustness of our novel bioinformatics approach. Furthermore, we were able to extend the current knowledge by discovering novel sites (cg26038589, cg10385208, cg09355027) with plausible connections to the diseases associated with smoking.

Another acknowledged issue is the missing gene annotations of measured CpG sites, which affects nearly every study on the effects of smoking on the DNA methylation, the majority conducted using the Illumina HM450 methylation array. In the present study, 16 significant CpG sites were not mapped to any gene, leaving the regulation potential and the participation in the biological pathways completely unknown. CpGs were mapped to gene sets via genes. CpGs mapping to any genomic region of the corresponding genes (for example, coding or promoter regions) were considered. As the pooling of CpGs sites from different regions of different genes was done, speculation on whether the altered methylation activates or deactivates gene expression is inconclusive and thus outside the scope of this work. However, it is important to note that function of DNA methylation varies with different genomic contexts [60].

The next logical step would be to include analysis based on the transcriptomics of the smoking- associated gene sets, elucidating the regulatory potential of the DNA methylation with respect to gene expression.

## Conclusions

The adverse health effects of smoking and the damage repair responses of the human body are presumably mediated by the epigenetic mechanisms regulating the gene expression. Extending previous works, the present study replicates 57 methylation sites and presents three novel sites (cg26038589, cg10385208, cg09355027) that potentially have roles in the cardiovascular disease, cancer, and immune response. As the most significant novel result, smoking alters methylation in the gene sets related to olfactory sensing system, which undergoes intense regeneration under tobacco smoke and toxin exposure. The other significant gene sets with smoking induced alteration in methylation related to cardiovascular disease, cancer, and immune response replicate the findings of the previous studies indicating the robustness of our novel bioinformatics analysis [26].

## Supplementary Material

Supplementary Tables S1-S2

## Abbreviations

BP: Biological Process
CC: Cellular Component
CGP: Chemical and Genetic Perturbations
CpG: 5′-Cytosine-phosphate-Guuanine-3′
FDR: False Discovery Rate
GO: Gene Ontology
KEGG: Kyoto Encyclopedia of Genes and Genomes
MF: Molecular Function
mGSZ: modified Gene Set *Z*-score
MSigDB: Molecular Signature Database
YFS: Young Finns Study

## References

[1] World Health Organization (2019) WHO report on the global tobacco epidemic. Retrieved from: https://www.who.int/tobacco/global_report/en/

[2] Talmud P.J., Stephens J.W., Hawe E., Demissie S., Cupples L.A., Hurel S.J. et al. (2005) The significant increase in cardiovascular disease risk in APOEɛ4 carriers is evident only in men who smoke: potential relationship between reduced antioxidant status and APOE4. Ann. Hum. Genet. 69, 613–622 10.1111/j.1529-8817.2005.00205.x 16266401

[3] Devereux T.R., Taylor J.A. and Barrett J.C. (1996) Molecular Mechanisms of Lung Cancer: Interaction of Environmental and Genetic Factors: Giles F. Filley Lecture Chest. 109, 14S–19S 8598134 10.1378/chest.109.3_supplement.14s

[4] Huan T., Joehanes R., Schurmann C., Schramm K., Pilling L.C., Peters M.J. et al. (2016) A whole-blood transcriptome meta-analysis identifies gene expression signatures of cigarette smoking. Hum. Mol. Genet. 25, 4611–4623 28158590 10.1093/hmg/ddw288 PMC5975607

[5] Feil R. and Fraga M.F. (2012) Epigenetics and the environment: emerging patterns and implications. Nat. Rev. Genet. 13, 97 10.1038/nrg3142 22215131

[6] Zeilinger S., Kühnel B., Klopp N., Baurecht H., Kleinschmidt A., Gieger C. et al. (2013) Tobacco smoking leads to extensive genome- wide changes in DNA methylation. PLoS ONE 8, e63812 10.1371/journal.pone.0063812 23691101 PMC3656907

[7] McCartney D.L., Stevenson A.J., Hillary R.F., Walker R.M., Bermingham M.L., Morris S.W. et al. (2018) Epigenetic signatures of starting and stopping smoking. EBioMedicine 37, 214–220 10.1016/j.ebiom.2018.10.051 30389506 PMC6286188

[8] Shenker N.S., Ueland P.M., Polidoro S., van Veldhoven K., Ricceri F., Brown R. et al. (2013) DNA methylation as a long-term biomarker of exposure to tobacco smoke. Epidemiology 712–716 10.1097/EDE.0b013e31829d5cb3 23867811

[9] Joehanes R., Just A.C., Marioni R.E., Pilling L.C., Reynolds L.M., Mandaviya P.R. et al. (2016) Epigenetic signatures of cigarette smoking. Circulation: Cardiovasc. Genet. 9, 436–447 27651444 10.1161/CIRCGENETICS.116.001506 PMC5267325

[10] Prince C., Hammerton G., Taylor A.E., Anderson E.L., Timpson N.J., Davey Smith G. et al. (2018) Investigating the impact of cigarette smoking behaviours on DNA methylation patterns in adolescence. Hum. Mol. Genet. 28, 155–165 10.1093/hmg/ddy316 PMC6298233 30215712

[11] Kaur G., Begum R., Thota S. and Batra S. (2019) A systematic review of smoking- related epigenetic alterations. Arch. Toxicol. 93, 1–26 31555878 10.1007/s00204-019-02562-y

[12] Ciccarone F., Tagliatesta S., Caiafa P. and Zampieri M. (2018) DNA methylation dynamics in aging: how far are we from understanding the mechanisms? Mechanisms of Ageing and Development, Elsevier Ireland Ltd 10.1016/j.mad.2017.12.002 29268958

[13] Yang Y., Gao X., Just A.C., Colicino E., Wang C., Coull B.A. et al. (2019) Smoking-related DNA methylation is associated with DNA methylation phenotypic age acceleration: The veterans affairs normative aging study. Int. J. Environ. Res. Public Health 16, 2356 10.3390/ijerph16132356 PMC6651499 31277270

[14] Lei M.K., Gibbons F.X., Simons R.L., Philibert R.A. and Beach S.R.H. (2020) The effect of tobacco smoking differs across indices of DNA methylation-based aging in an african american sample: DNA methylation-based indices of smoking capture these effects. Genes 11, 311 10.3390/genes11030311 PMC7140795 32183340

[15] Ashburner M., Ball C.A., Blake J.A., Botstein D., Butler H., Cherry J.M. et al. (2000) Gene ontology: tool for the unification of biology. Nat. Genet. 25, 25 10.1038/75556 10802651 PMC3037419

[16] Rotroff D.M., Joubert B.R., Marvel S.W., Håberg S.E., Wu M.C., Nilsen R.M. et al. (2016) Maternal smoking impacts key biological pathways in newborns through epigenetic modification in Utero. BMC Genomics 17, 976 10.1186/s12864-016-3310-1 27887572 PMC5124223

[17] Bakulski K.M., Dou J., Lin N., London S.J. and Colacino J.A. (2019) DNA methylation signature of smoking in lung cancer is enriched for exposure signatures in newborn and adult blood. Sci. Rep. 9, 1–13 10.1038/s41598-019-40963-2 30872662 PMC6418160

[18] Fabregat A., Jupe S., Matthews L., Sidiropoulos K., Gillespie M., Garapati P. et al. (2017) The reactome pathway knowledgebase. Nucleic Acids Res. 46, D649–D655 10.1093/nar/gkx1132 PMC5753187 29145629

[19] Nishimura D.B. Biotech Software & Internet Report: The Computer Software Journal for Scient. 2, 117–120

[20] Ogata H., Goto S., Sato K., Fujibuchi W., Bono H. and Kanehisa M. (1999) KEGG: Kyoto encyclopedia of genes and genomes. Nucleic Acids Res. 27, 29–34 10.1093/nar/27.1.29 9847135 PMC148090

[21] Qiu W., Wan E., Morrow J., Cho M.H., Crapo J.D., Silverman E.K. et al. (2015) The impact of genetic variation and cigarette smoke on DNA methylation in current and former smokers from the COPDGene study. Epigenetics 10, 1064–1073 10.1080/15592294.2015.1106672 26646902 PMC4844199

[22] Goeman J.J. and Bühlmann P. (2007) Analyzing gene expression data in terms of gene sets: methodological issues. Bioinformatics 23, 980–987 10.1093/bioinformatics/btm051 17303618

[23] Subramanian A., Tamayo P., Mootha V.K., Mukherjee S., Ebert B.L., Gillette M.A. et al. (2005) Gene set enrichment analysis: a knowledge-based approach for interpreting genome-wide expression profiles. Proc. Natl. Acad. Sci. U.S.A. 102, 15545–15550 10.1073/pnas.0506580102 16199517 PMC1239896

[24] Liberzon A., Subramanian A., Pinchback R., Thorvaldsdóttir H., Tamayo P. and Mesirov J.P. (2011) Molecular signatures database (MSigDB) 3.0. Bioinformatics 27, 1739–1740 10.1093/bioinformatics/btr260 21546393 PMC3106198

[25] Törönen P., Ojala P.J., Marttinen P. and Holm L. (2009) Robust extraction of functional signals from gene set analysis using a generalized threshold free scoring function. BMC Bioinformatics 10, 307 10.1186/1471-2105-10-307 19775443 PMC2761411

[26] Mishra P., Törönen P., Leino Y. and Holm L. (2014) Gene set analysis: limitations in popular existing methods and proposed improvements. Bioinformatics 30, 2747–2756 10.1093/bioinformatics/btu374 24903419

[27] Mishra P.P., Medlar A., Holm L. and Törönen P. (2016) Robust multi- group gene set analysis with few replicates. BMC Bioinformatics 17, 526 10.1186/s12859-016-1403-0 27938331 PMC5148902

[28] Naeem H., Zimmer R., Tavakkolkhah P. and Küffner R. (2012) Rigorous assessment of gene set enrichment tests. Bioinformatics 28, 1480–1486 10.1093/bioinformatics/bts164 22492315

[29] Knijnenburg T.A., Wessels L. F.A., Reinders M. J.T. and Shmulevich I. (2009) Fewer permutations, more accurate P-values. Bioinformatics 25, 1161–1168 10.1093/bioinformatics/btp211 19477983 PMC2687965

[30] Ajmani G.S., Suh H.H., Wroblewski K.E. and Pinto J.M. (2017) Smoking and olfactory dysfunction: A systematic literature review and meta-analysis. Laryngoscope 127, 1753–1761 10.1002/lary.26558 28561327 PMC6731037

[31] Raitakari O.T., Juonala M., Rönnemaa T., Keltikangas-Järvinen L., Räsänen L., Pietikäinen M. et al. (2008) Cohort profile: the cardiovascular risk in Young Finns Study. Int. J. Epidemiol. 37, 1220–1226 10.1093/ije/dym225 18263651

[32] Bibikova M., Barnes B., Tsan C., Ho V., Klotzle B., Le J.M. et al. (2011) High density DNA methylation array with single CpG site resolution. Genomics 98, 288–295 10.1016/j.ygeno.2011.07.007 21839163

[33] Aryee M.J., Jaffe A.E., Corrada-Bravo H., Ladd-Acosta C., Feinberg A.P., Hansen K.D. et al. (2014) Minfi: a flexible and comprehensive Bioconductor package for the analysis of Infinium DNA methylation microarrays. Bioinformatics 30, 1363–1369 10.1093/bioinformatics/btu049 24478339 PMC4016708

[34] Triche T.J. Jr, Weisenberger D.J., Van Den Berg D., Laird P.W. and Siegmund K.D. (2013) Low-level processing of Illumina Infinium DNA methylation beadarrays. Nucleic Acids Res. 41, e90 10.1093/nar/gkt090 23476028 PMC3627582

[35] Team R.C. R: A language and environment for statistical computing. URL https://www.R-project.org/

[36] Barfield R.T., Kilaru V., Smith A.K. and Conneely K.N. (2012) CpGassoc: an R function for analysis of DNA methylation microarray data. Bioinformatics 28, 1280–1281 10.1093/bioinformatics/bts124 22451269 PMC3577110

[37] Wilson L.E., Xu Z., Harlid S., White A.J., Troester M.A., Sandler D.P. et al. (2019) Alcohol and DNA Methylation: An Epigenome-Wide Association Study in Blood and Normal Breast Tissue. Am. J. Epidemiol. 188, 1055–1065 10.1093/aje/kwz032 30938765 PMC6545285

[38] McDade T.W., Ryan C.P., Jones M.J., Hoke M.K., Borja J., Miller G.E. et al. (2019) Genome-wide analysis of DNA methylation in relation to socioeconomic status during development and early adulthood. Am. J. Phys. Anthropol. 169, 3–11 10.1002/ajpa.23800 30771258

[39] Magnussen C.G., Venn A., Thomson R., Juonala M., Srinivasan S.R., Viikari J.S. et al. (2009) The association of pediatric low-and high-density lipoprotein cholesterol dyslipidemia classifications and change in dyslipidemia status with carotid intima-media thickness in adulthood: evidence from the Cardiovascular Risk in Young Finns Study, the Bogalusa Heart Study, and the CDAH (Childhood Determinants of Adult Health) Study. J. Am. Coll. Cardiol. 53, 860–869 19264243 10.1016/j.jacc.2008.09.061 PMC2759186

[40] Houseman E.A., Accomando W.P., Koestler D.C., Christensen B.C., Marsit C.J., Nelson H.H. et al. (2012) DNA methylation arrays as surrogate measures of cell mixture distribution. BMC Bioinformatics 13, 86 10.1186/1471-2105-13-86 22568884 PMC3532182

[41] IlluminaHumanMethylation450kanno HK (2016) ilmn12. hg19: annotation for illumina's 450k methylation arrays. R package version 0.6.0

[42] Beißbarth T. and Speed T.P. (2004) GOstat: find statistically overrepresented Gene Ontologies within a group of genes. Bioinformatics 20, 1464–1465 10.1093/bioinformatics/bth088 14962934

[43] Alexa A. and Rahnenführer J. (2009) Gene set enrichment analysis with topGO. Bioconductor Improv. 27

[44] Liberzon A., Birger C., Thorvaldsdóttir H., Ghandi M., Mesirov J.P. and Tamayo P. (2015) The molecular signatures database hallmark gene set collection. Cell Syst. 1, 417–425 10.1016/j.cels.2015.12.004 26771021 PMC4707969

[45] Shi Q., Bao S., Song L., Wu Q., Bigner D.D., Hjelmeland A.B. et al. (2007) Targeting SPARC expression decreases glioma cellular survival and invasion associated with reduced activities of FAK and ILK kinases. Oncogene 26, 4084 10.1038/sj.onc.1210181 17213807

[46] Su D., Wang X., Campbell M.R., Porter D.K., Pittman G.S., Bennett B.D. et al. (2016) Distinct epigenetic effects of tobacco smoking in whole blood and among leukocyte subtypes. PLoS ONE 11, e0166486 10.1371/journal.pone.0166486 27935972 PMC5147832

[47] Ringh M.V., Hagemann-Jensen M., Needhamsen M., Kular L., Breeze C.E., Sjöholm L.K. et al. (2019) Tobacco smoking induces changes in true DNA methylation, hydroxymethylation and gene expression in bronchoalveolar lavage cells. EBioMedicine 46, 290–304 10.1016/j.ebiom.2019.07.006 31303497 PMC6710853

[48] Galganski L., Urbanek M.O. and Krzyzosiak W.J. (2017) Nuclear speckles: molecular organization, biological function and role in disease. Nucleic Acids Res. 45, 10350–10368 10.1093/nar/gkx759 28977640 PMC5737799

[49] Fishilevich S., Zimmerman S., Kohn A., Iny Stein T., Olender T., Kolker E. et al. (2016) Genic insights from integrated human proteomics in GeneCards. Database 2016, 10.1093/database/baw030 27048349 PMC4820835

[50] Nazarian A., Arbeev K.G., Yashkin A.P. and Kulminski A.M. (2019) Genetic heterogeneity of Alzheimer's disease in subjects with and without hypertension. GeroScience 41, 137–154 10.1007/s11357-019-00071-5 31055733 PMC6544706

[51] Stirzaker C., Song J.Z., Ng W., Du Q., Armstrong N.J., Locke W.J. et al. (2017) Methyl-CpG-binding protein MBD2 plays a key role in maintenance and spread of DNA methylation at CpG islands and shores in cancer. Oncogene 36, 1328 10.1038/onc.2016.297 27593931

[52] Rappaport N., Twik M., Plaschkes I., Nudel R., Iny Stein T., Levitt J. et al. (2016) MalaCards: an amalgamated human disease compendium with diverse clinical and genetic annotation and structured search. Nucleic Acids Res. 45, D877–D887 10.1093/nar/gkw1012 27899610 PMC5210521

[53] Yee K.K., Pribitkin E.A., Cowart B.J., Vainius A.A., Klock C.T., Rosen D. et al. (2009) Smoking-associated squamous metaplasia in olfactory mucosa of patients with chronic rhinosinusitis. Toxicol. Pathol. 37, 594–598 10.1177/0192623309338055 19487255 PMC5898236

[54] Choi R. and Goldstein B.J. (2018) Olfactory epithelium: cells, clinical disorders, and insights from an adult stem cell niche. Laryngoscope Invest. Otolaryngol. 3, 35–42 10.1002/lio2.135 29492466 PMC5824112

[55] Goldstein B.J., Goss G.M., Choi R., Saur D., Seidler B., Hare J.M. et al. (2016) Contribution of Polycomb group proteins to olfactory basal stem cell self-renewal in a novel c-KIT+ culture model and in vivo. Development 143, 4394–4404 10.1242/dev.142653 27789621 PMC5201051

[56] Suarez L., Ramadhani T., Felkner M., Canfield M.A., Brender J.D., Romitti P.A. et al. (2011) Maternal smoking, passive tobacco smoke, and neural tube defects. Birth Defects Res. Part A: Clin. and Mol. Teratol. 91, 29–33 10.1002/bdra.20743 21254356 PMC6034638

[57] Wang D. (2018) The essential role of G protein-coupled receptor (GPCR) signaling in regulating T cell immunity. Immunopharmacol. Immunotoxicol. 40, 187–192 10.1080/08923973.2018.1434792 29433403

[58] Coughlin S.R. (2000) Thrombin signalling and protease-activated receptors. Nature 407, 258 10.1038/35025229 11001069

[59] Li H.X., Peng X.X., Zong Q., Zhang K., Wang M.X., Liu Y.Z. et al. (2016) Cigarette smoking and risk of adult glioma: a meta-analysis of 24 observational studies involving more than 2.3 million individuals. OncoTargets Ther. 9, 3511 27366088 10.2147/OTT.S99713 PMC4913539

[60] Jones P.A. (2012) Functions of DNA methylation: islands, start sites, gene bodies and beyond. Nat. Rev. Genet. 13, 484 10.1038/nrg3230 22641018

